# Pain Intervention for Cancer and Non-cancer Pain: A Retrospective Analysis of Tertiary Care Hospital Experience

**DOI:** 10.7759/cureus.7719

**Published:** 2020-04-18

**Authors:** Umair Ahmad, Syed A Abbas, Syeda M Hamadani, Syed M Abbas, Samia Usman, Zeeshan Hafeez, Ateeq Ur Rehman Ghafoor

**Affiliations:** 1 Anaesthesia, Shaukat Khanum Memorial Cancer Hospital and Research Center, Lahore, PAK; 2 Internal Medicine, Ittefaq Trust Hospital, Lahore, PAK; 3 Internal Medicine, Montefiore Medical Center/Albert Einstein College of Medicine, Bronx, USA; 4 Internal Medicine, Fatima Memorial Hospital College of Medicine and Dentistry, Lahore, PAK; 5 Internal Medicine, Montefiore Medical Center, Wakefield Campus, Bronx, USA; 6 Internal Medicine, King Edward Medical College, Lahore, PAK; 7 Internal Medicine, Westchester Medical Center, Valhalla, USA; 8 Anesthesia and Pain Management, Shaukat Khanum Memorial Cancer Hospital and Research Centre, Lahore, PAK

**Keywords:** chronic pain, pain management, cancer and non cancer pain

## Abstract

Background

With the recent advancement in medicine there has been a great emphasis on the management of chronic pain which remains as one of the major contributing factors for functional limitation in patients as well as a financial burden on healthcare. Newer treatment modalities are aimed at terminating the vicious pain cycles and in this regard peripheral nerve blocks have proven to be very effective.

Objectives

The aim of this study is to evaluate the effectiveness of interventions for both cancer and non-cancer patients by objective assessment of the patients before and after the procedure.

Materials and methods

The study included 252 patients who underwent nerve block procedures in Shaukat Khanum Memorial Cancer Hospital from December 2016 to December 2018. The patients were evaluated using numerical rating scale (NRS) for pain, reduction in analgesic doses and patient satisfaction after one and four weeks post procedure. The data was analyzed using mean values and calculating percentages.

Results

In cancer group, 168 patients were included; mean age 50.49 ± 15.39 with 46.43% females and 53.57% males, the average pain score was 2.62 ± 1.87 post procedure compared with 6.30 ± 1.87 post procedure. 48.21% of the patients reported a reduction in analgesia while 51.79% of the patients kept on using the same analgesics doses. 74.40% of the patients were satisfied and 25.60% patients remained unsatisfied after one week whereas 66.07% were satisfied, 23.81% were not satisfied and 10.12% loss to follow up after four weeks. In non-cancer group 84 patients were included; mean age 56.49 ± 15.79 with 41.67% females and 58.33% males, the average pain score before intervention was 5.99 ± 1.21 and after intervention it was 2.43 ± 1.62. In 73.81% non-cancer patients the analgesics doses were reduced and 70.24% patients were satisfied while 29.76% were unsatisfied after one week. After four weeks 55.95% were satisfied, 22.62% were not satisfied and 21.43% loss to follow up.

Conclusion

The study showed decrease in pain scores in both group of patients and the importance of nerve blocks as an effective method for chronic pain management. The reduction in the use of other analgesics was also commendable in both the groups.

## Introduction

With the recent advancement in medicine there has been a great emphasis on the management of chronic pain which remains as one of the major contributing factors for functional limitation in patients and poor quality of life as well as a financial burden on healthcare. The prevalence of chronic pain is estimated to be 37.3% in developed and 41.1% in developing countries [1].

In 2012, there were more than 14 million diagnosed cancer patients and it is estimated that it will rise to more than 20 million by 2025 [2]. With better treatment options more patients are surviving with cancer and one of the fearsome aspects of this disease is chronic pain which even at present is very challenging to manage. Although the use of opioids has greatly helped in reducing the pain associated with the disease but still the prevalence of chronic pain remains high.

Similarly non-cancer chronic pain is very common and its prevalence was estimated to be 19% in Europe [3]. The American Pain Society survey also has estimated that 9% of the adult population suffers from moderate to severe, non-cancer related pain [4]. Epidemiological data in the elderly population estimates that up to 50% of them suffer from chronic pain [5].

In the past few decades, the mainstay management for chronic pain was opioids and topical therapies. World Health Organization (WHO) also recommends use of opioids as part of the analgesic step-ladder approach but prolonged use of opioid medication is associated with serious side effects and patient compliance is an issue. Recently, researchers are focused towards discovering the changes that happen in brain and nervous system because of chronic pain to better understand the physical basis and to establish better treatment options. Newer treatment modalities are aimed at terminating the vicious pain cycles and in this regard nerve blocks have proven to be very effective.

Previously an abstract was presented which showed the efficacy of interventional pain procedures for cancer and non-cancer pain (Poster presentation: Ahmad U. Pain Interventions for Cancer and Non-Cancer Pain: A Retrospective Analysis of Shaukat Khanum Memorial Cancer Hospital and Research Centre Experience. 17th Shaukat Khanum Cancer Symposium; Nov 2-4, 2018). The primary objective of this study is to emphasize the role of interventional procedures along with the pharmacological therapy in pain management.

## Materials and methods

**Figure 1:**
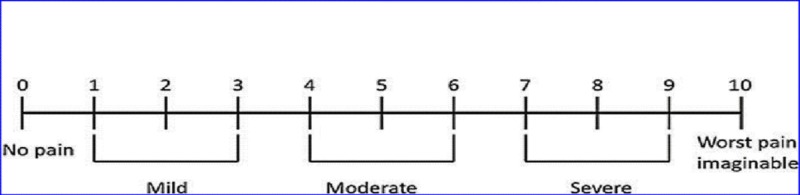
Numerical Rating Scale (NRS)

In this study, retrospective data of patients was analyzed who underwent interventional pain procedures at Shaukat Khanum Memorial Cancer Hospital and Research Centre from December 2016 to December 2018. The data was collected after getting Institutional Review Board (IRB) approval. The patients were evaluated using numerical rating scale (NRS) for pain (Figure 1), reduction in analgesic doses and patient satisfaction after one and four weeks post procedure. The patient satisfaction criteria were based on reduction in pain score according to NRS and the improvement in carrying out daily routine activities (Tables 1, 2). The data was analyzed using mean values and calculating percentages.

**Table 1:** Pain Score Table

Pre Procedure Pain Score									
1	2	3	4	5	6	7	8	9	10

Post Procedure Pain Score									
1	2	3	4	5	6	7	8	9	10

**Table 2:** Satisfaction Score Table

Would you undergo repeat procedure if required?	Yes	No
Do you feel more comfortable in carrying out daily activities post procedure?	Yes	No
Do you have to wake up at night due to pain post procedure?	Yes	No
Do you feel more refreshed in the morning post procedure?	Yes	No
Do you feel less need of pain medications post procedure?	Yes	No
Would you recommend the procedure to your family/friends for pain control?	Yes	No

## Results

The study included 252 patients which were further divided into two groups: cancer and non-cancer patients. In cancer group 168 patients were included; their mean age was 50.49 ± 15.39 with 46.43% females and 53.57% males, the average pain score was 2.62 ± 1.87 after the procedure compared with 6.30 ± 1.87 before the intervention. 48.21% of the patients reported a reduction in analgesic doses while 51.79% of the patients kept on using the same analgesics doses. 74.40% of the patients were satisfied and 25.60% patients remained unsatisfied after one week whereas 66.07% were satisfied, 23.81% were not satisfied and 10.12% loss to follow up after four weeks (Tables 3-6).

**Table 3:** Pain Interventional Procedures in Cancer Patients NRS: Numerical pain score

Total number of patients	168
Pain score before procedure	6.30 (NRS)
Pain score after procedure	2.62 (NRS)
Reduction in analgesic medication dose reported by patients	81
Overall Satisfaction after 1 week	74.40%
Overall Satisfaction after 4 weeks	66.07%

**Table 4:** Coeliac Plexus Block in Cancer Patients NRS: Numerical pain score

Total number of patients	40
Pain score before procedure	7.1 (NRS)
Pain score after procedure	2.8 (NRS)
Reduction in analgesic medication dose reported by patients	25
Overall Satisfaction after 1 week	75%
Overall Satisfaction after 4 weeks	55%

**Table 5:** Epidural Rhizolysis in Cancer Patients NRS: Numerical pain score

Total number of patients	40
Pain score before procedure	5.8 (NRS)
Pain score after procedure	2.3 (NRS)
Reduction in analgesic medication dose reported by patients	19
Overall Satisfaction after 1 week	80%
Overall Satisfaction after 4 weeks	75%

**Table 6:** Intrathecal Neurolysis in Cancer Patients NRS: Numerical pain score

Total number of patients	30
Pain score before procedure	5.5 (NRS)
Pain score after procedure	2.7 (NRS)
Reduction in analgesic medication dose reported by patients	5
Overall Satisfaction after 1 week	70%
Overall Satisfaction after 4 weeks	60%

In non-cancer group 84 patients were included with mean age of 56.49 ± 15.79 with 41.67% females and 58.33% males, the average pain score before intervention was 5.99 ± 1.21 and after intervention it was 2.43 ± 1.62. In 73.81% non-cancer patients the analgesics doses were reduced and 70.24% patients were satisfied while 29.76% were unsatisfied after one week. After four weeks 55.95% were satisfied, 22.62% were not satisfied and 21.43% loss to follow up (Tables 7-10).

**Table 7:** Pain Interventional Procedures in Non-Cancer Patients NRS: Numerical pain score

Total number of patients	84
Pain score before procedure	5.99 (NRS)
Pain score after procedure	2.43 (NRS)
Reduction in analgesic medication dose reported by patients	62
Overall Satisfaction after 1 week	70.24%
Overall Satisfaction after 4 weeks	55.95%

**Table 8:** Intra-articular Injection in Non-Cancer Patients NRS: Numerical pain score

Total number of patients	32
Pain score before procedure	6.0 (NRS)
Pain score after procedure	2.5 (NRS)
Reduction in analgesic medication dose reported by patients	22
Overall Satisfaction after 1 week	62.5%
Overall Satisfaction after 4 weeks	56.25%

**Table 9:** Epidural Rhizolysis in Non-Cancer Patients NRS: Numerical pain score

Total number of patients	32
Pain score before procedure	6.2 (NRS)
Pain score after procedure	2.4 (NRS)
Reduction in analgesic medication dose reported by patients	26
Overall Satisfaction after 1 week	81.25%
Overall Satisfaction after 4 weeks	62.5%

**Table 10:** Ganglion Impar Block in Non-Cancer Patients NRS: Numerical pain score

Total number of patients	6
Pain score before procedure	6.8 (NRS)
Pain score after procedure	2.4 (NRS)
Reduction in analgesic medication dose reported by patients	3
Overall Satisfaction after 1 week	66.66%
Overall Satisfaction after 4 weeks	50%

## Discussion

Chronic pain remains as one of the major factors that has a negative impact on patient’s physical and psychological health. Chronic pain not only adversely affects the patient but also their families. The WHO analgesic ladder provides the basic guidelines to address chronic pain depending on the disease severity. Most of the patients are on opioids for pain control with variable tolerance to medication. Although opioids provide good pain relief for these patients, but opioids do have side effects and if not managed properly they can be a reason for non-compliance and poor quality of life. Also, with the prolonged use there are tolerance and dependency issues. Nerve blocks have been used for pain management for over a century now. In 1884, Koller first reported the use of nerve blocks [6]. They can be used either with local anesthetics or neurolytic agents. Nerve blocks act by inhibiting the impulse transmission from the peripheral nerve ending resulting in termination of the pain signal perceived by the cortex.

In this study, we have evaluated the efficacy of nerve blocks in chronic pain management, reduction in analgesic doses and patient satisfaction post procedure. The study included 252 patients out of which 168 are cancer patients and 84 non-cancer patients. The patients underwent different pain interventional procedures and numerical pain scores were used to assess the efficacy of the treatment.

In cancer patients, most of the procedures were carried out with palliative intent to control the pain and improve the quality of life in terminal patients. Few of the patients have to undergo repeat procedures for adequate pain control. The prevalence of pain in cancer patients with advanced stage is around 62%-86% which emphasize that adequate pain control is not achieved in majority of the patients [7-10].

Most common procedure carried out in our centre was coeliac plexus block (40), epidural rhizolysis (40) and intrathecal neurolysis (30) in cancer patients. Coeliac plexus block is carried out in patients with intractable pain in pancreatic and upper abdominal organ carcinomas. Recently a study was published which emphasized on the efficacy of these blocks for upper abdominal cancer pain [11]. Most of the patients reported adequate pain control with the intervention as documented in previous studies [9, 12, 13]. The requirement of other analgesic medications was not significantly decreased in these patients. Few of the patients had to undergo a repeat procedure because of the underlying disease progression for adequate pain control [14].

In epidural rhizolysis the nerves carrying sensation to the spinal cord are desensitized by using a combination of local anaesthetic and steroid so that pain sensation is reduced. Epidural injections have been carried out since 1900s for relieving back pain. The efficacy of this procedure in reducing pain is well established in patients with refractory cancer pain [15, 16]. At our centre a total of 59 epidural rhizolysis procedures were carried out both for cancer and non-cancer pain at different spinal levels. There have been debates regarding efficacy and complications related to epidural injections but in our centre majority of the patients reported good pain relief post procedure.

The procedure for intrathecal neurolysis was first described by Dogliotti in 1931 and has been used since then for intractable cancer pain. Careful selection of the patients is needed as some serious complications are associated with the procedure. The chemical neurolysis had been carried out with different agents including alcohol and phenol-glycerol combination with similar pain relief results in patients [17-19]. Likewise with other pain interventional procedures there was a significant reduction in pain scores as analyzed by the numerical pain score (NRS) and around 74.40% patients were satisfied in terms of pain control after one week post procedure.

For non-cancer pain intra-articular injection (32), epidural rhizolysis (32) and ganglion impar blocks (6) were the most common procedures. Musculoskeletal pain is the most common type of chronic pain seen in the adult population. In US adults the prevalence of doctor-diagnosed arthritis was 21% (46.4 million persons) [20]. The pain due to joint disease is among the top 10 causes of disability worldwide [21]. Intra-articular corticosteroid and hyaluronic acid injections are used if the pharmacological therapy is not effective.

Ganglion impar block (GIB) was first described in 1990 and was primarily used for pain control in cancer patients. Since then the procedure is commonly performed for pain in the terminal segment of the spine near the coccyx and perineal area also referred to as Coccydynia. The blockade of nociceptive and sympathetic fibers is achieved by this block which helps in pain relief [22]. Various methods and techniques have been described for this procedure. A number of studies have been conducted to show the efficacy and safety of the block in relieving perineal pain [22-24]. In non-cancer group 73.81% reported reduction in analgesic doses as compared to cancer group which showed only 48.21%. Although the patient satisfaction after one week post procedure was similar in both the groups.

## Conclusions

Pain interventional procedures showed much better pain control and patient satisfaction in both cancer and non-cancer group as compared to conventional pharmacological therapy. Interventional procedures can be used as an adjuvant to pharmacological therapy and also will help in reducing opioid dose and their side effects.
